# 2-Chloro-5-({[5-(4-meth­oxy­phen­yl)-1,3,4-oxadiazol-2-yl]­sulfanyl}­meth­yl)pyridine

**DOI:** 10.1107/S1600536811050410

**Published:** 2011-11-30

**Authors:** Hao Ji, Xu-Dong Xu

**Affiliations:** a Department of Bioengineering, College of Medicine, Southeast University, Nanjing 210009, People’s Republic of China, and Jiangsu Tiansheng Pharmaceutical, Company Limited Jurong City 212415, Jiangsu Province, People’s Republic of China

## Abstract

In the title compound, C_15_H_12_ClN_3_O_2_S, the central oxadiazole ring forms dihedral angles of 7.72 (14) and 69.86 (12)° with the benzene and pyridine rings, respectively. The crystal packing is governed only by van der Waals inter­actions.

## Related literature

For background to the biological activity of heterocyclic compounds, see: Mamolo *et al.* (2001 ▶); Liu *et al.* (2001 ▶); Demirbas *et al.* (2004 ▶). For the synthesis, see: Zareef *et al.* (2008 ▶); Wu *et al.* (2011 ▶). For standard bond lengths, see: Allen *et al.* (1987 ▶). 
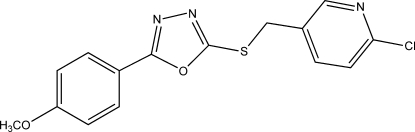


## Experimental

### 

#### Crystal data

C_15_H_12_ClN_3_O_2_S
*M* *_r_* = 333.80
Orthorhombic, 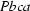

*a* = 12.311 (2) Å
*b* = 8.1229 (15) Å
*c* = 29.956 (6) Å
*V* = 2995.6 (10) Å^3^
*Z* = 8
Mo *K*α radiation
μ = 0.40 mm^−1^
*T* = 298 K
0.30 × 0.20 × 0.05 mm

#### Data collection

Bruker SMART APEX area-detector diffractometer
Absorption correction: multi-scan (*SADABS*; Sheldrick, 1996 ▶) *T*_min_ = 0.886, *T*_max_ = 0.980
5300 measured reflections
2730 independent reflections
1514 reflections with *I* > 2σ(*I*)
*R*_int_ = 0.089

#### Refinement

*R*[*F* ^2^ > 2σ(*F* ^2^)] = 0.060
*wR*(*F* ^2^) = 0.147
*S* = 0.97
2730 reflections
201 parameters
H-atom parameters constrained
Δρ_max_ = 0.24 e Å^−3^
Δρ_min_ = −0.23 e Å^−3^

### 

Data collection: *SMART* (Bruker, 1998 ▶); cell refinement: *SAINT* (Bruker, 1998 ▶); data reduction: *SAINT*; program(s) used to solve structure: *SHELXS97* (Sheldrick, 2008 ▶); program(s) used to refine structure: *SHELXL97* (Sheldrick, 2008 ▶); molecular graphics: *SHELXTL* (Sheldrick, 2008 ▶); software used to prepare material for publication: *SHELXTL*.

## Supplementary Material

Crystal structure: contains datablock(s) global, I. DOI: 10.1107/S1600536811050410/rz2672sup1.cif

Structure factors: contains datablock(s) I. DOI: 10.1107/S1600536811050410/rz2672Isup2.hkl

Supplementary material file. DOI: 10.1107/S1600536811050410/rz2672Isup3.cml

Additional supplementary materials:  crystallographic information; 3D view; checkCIF report

## Figures and Tables

**Fig. 1.**
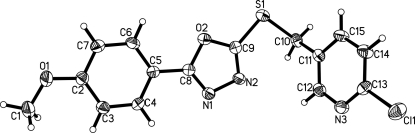
The molecular structure of the title compound showing 30% probability displacement ellipsoids.

## supplementary crystallographic information

### Comment

Heterocyclic compounds have been of great interest since many years, in particular due to the important role these compouns play in the development of medicinal chemistry (Mamolo *et al.*, 2001; Liu *et al.*, 2001; Demirbas *et al.*, 2004). As a contribution to the structural characterization of new heterocyclic compounds, we report here the structure of the title compound.

In the title compound (Fig. 1) all bond lengths are within normal ranges (Allen *et al.*, 1987). The dihedral angle between the central oxadiazole ring (N1/N2/O2/C8/C9) and the benzene (C2–C7) and pyridine (N3/C11–C15) rings are of 7.72 (14) and 69.86 (12)°, respectively. In the crystal structure, no hydrogen bonds, π···π interactions or C—H···π short contacts are observed, the structure being stabilized only by van der Waals interactions.

### Experimental

The title compound was synthesized according to the previously reported literature methods (Zareef *et al.*, 2008; Wu *et al.*, 2011). Single crystals suitable for X-ray diffraction analysis were obtained by evaporation of an ethanol solution.

### Refinement

All H atoms were placed in geometrically idealized positions and constrained to ride on their parent atoms, with C—H distances of 0.93–0.97 Å, and with with *U*_iso_(H) = 1.2*U*_eq_(C) or 1.5*U*_eq_(C) for methyl H atoms.

### Crystal data

**Table:**

C_15_H_12_ClN_3_O_2_S	*F*(000) = 1376
*M**_r_* = 333.80	*D*_x_ = 1.480 Mg m^−^^3^
Orthorhombic, *P**b**c**a*	Mo *K*α radiation, λ = 0.71073 Å
Hall symbol: -P 2ac 2ab	Cell parameters from 2256 reflections
*a* = 12.311 (2) Å	θ = 4.2–26°
*b* = 8.1229 (15) Å	µ = 0.40 mm^−^^1^
*c* = 29.956 (6) Å	*T* = 298 K
*V* = 2995.6 (10) Å^3^	Needle, yellow
*Z* = 8	0.30 × 0.20 × 0.05 mm

### Data collection

**Table:**

Bruker SMART APEX area-detector diffractometer	2730 independent reflections
Radiation source: fine-focus sealed tube	1514 reflections with *I* > 2σ(*I*)
graphite	*R*_int_ = 0.089
ω scans	θ_max_ = 25.3°, θ_min_ = 1.4°
Absorption correction: multi-scan (*SADABS*; Sheldrick, 1996)	*h* = −14→14
*T*_min_ = 0.886, *T*_max_ = 0.980	*k* = −9→0
5300 measured reflections	*l* = 0→35

### Refinement

**Table:**

Refinement on *F*^2^	Primary atom site location: structure-invariant direct methods
Least-squares matrix: full	Secondary atom site location: difference Fourier map
*R*[*F*^2^ > 2σ(*F*^2^)] = 0.060	Hydrogen site location: inferred from neighbouring sites
*wR*(*F*^2^) = 0.147	H-atom parameters constrained
*S* = 0.97	*w* = 1/[σ^2^(*F*_o_^2^) + (0.0512*P*)^2^] where *P* = (*F*_o_^2^ + 2*F*_c_^2^)/3
2730 reflections	(Δ/σ)_max_ < 0.001
201 parameters	Δρ_max_ = 0.24 e Å^−^^3^
0 restraints	Δρ_min_ = −0.23 e Å^−^^3^

### Special details

**Table:**

Geometry. All e.s.d.'s (except the e.s.d. in the dihedral angle between two l.s. planes) are estimated using the full covariance matrix. The cell e.s.d.'s are taken into account individually in the estimation of e.s.d.'s in distances, angles and torsion angles; correlations between e.s.d.'s in cell parameters are only used when they are defined by crystal symmetry. An approximate (isotropic) treatment of cell e.s.d.'s is used for estimating e.s.d.'s involving l.s. planes.
Refinement. Refinement of *F*^2^ against ALL reflections. The weighted *R*-factor *wR* and goodness of fit *S* are based on *F*^2^, conventional *R*-factors *R* are based on *F*, with *F* set to zero for negative *F*^2^. The threshold expression of *F*^2^ > σ(*F*^2^) is used only for calculating *R*-factors(gt) *etc*. and is not relevant to the choice of reflections for refinement. *R*-factors based on *F*^2^ are statistically about twice as large as those based on *F*, and *R*- factors based on ALL data will be even larger.

### Fractional atomic coordinates and isotropic or equivalent isotropic displacement parameters (Å^2^)

**Table:**

	*x*	*y*	*z*	*U*_iso_*/*U*_eq_	
C1	0.5087 (4)	−0.2986 (7)	0.24553 (18)	0.0714 (16)	
H1A	0.5338	−0.1867	0.2461	0.107*	
H1B	0.5102	−0.3387	0.2154	0.107*	
H1C	0.5552	−0.3654	0.2638	0.107*	
C2	0.3779 (4)	−0.2211 (6)	0.29959 (14)	0.0472 (11)	
C3	0.4536 (3)	−0.1655 (5)	0.33013 (14)	0.0455 (11)	
H3	0.5271	−0.1861	0.3256	0.055*	
C4	0.4201 (3)	−0.0794 (5)	0.36728 (13)	0.0420 (11)	
H4	0.4715	−0.0414	0.3876	0.050*	
C5	0.3108 (3)	−0.0486 (5)	0.37482 (13)	0.0371 (10)	
C6	0.2359 (3)	−0.1103 (6)	0.34482 (14)	0.0507 (12)	
H6	0.1621	−0.0936	0.3498	0.061*	
C7	0.2693 (4)	−0.1962 (6)	0.30773 (15)	0.0600 (14)	
H7	0.2179	−0.2379	0.2880	0.072*	
C8	0.2797 (3)	0.0489 (5)	0.41334 (13)	0.0364 (10)	
C9	0.1752 (3)	0.1674 (5)	0.46014 (14)	0.0396 (11)	
C10	0.1054 (4)	0.3455 (5)	0.52966 (12)	0.0442 (11)	
H10A	0.1658	0.4113	0.5190	0.053*	
H10B	0.0493	0.4206	0.5397	0.053*	
C11	0.1431 (3)	0.2446 (5)	0.56864 (13)	0.0384 (10)	
C12	0.2518 (3)	0.2350 (6)	0.57983 (14)	0.0473 (11)	
H12	0.3018	0.2869	0.5613	0.057*	
C13	0.2170 (4)	0.0810 (6)	0.64102 (14)	0.0454 (12)	
C14	0.1079 (4)	0.0776 (6)	0.63222 (15)	0.0518 (12)	
H14	0.0604	0.0206	0.6507	0.062*	
C15	0.0706 (3)	0.1604 (6)	0.59533 (14)	0.0481 (12)	
H15	−0.0030	0.1597	0.5884	0.058*	
N1	0.3412 (3)	0.1276 (4)	0.44087 (11)	0.0436 (9)	
N2	0.2721 (3)	0.2060 (5)	0.47184 (11)	0.0447 (9)	
N3	0.2897 (3)	0.1545 (5)	0.61608 (12)	0.0501 (10)	
O1	0.4021 (3)	−0.3058 (5)	0.26198 (11)	0.0770 (12)	
O2	0.1718 (2)	0.0681 (3)	0.42357 (9)	0.0415 (7)	
S	0.05258 (9)	0.22470 (15)	0.48350 (4)	0.0480 (3)	
Cl1	0.26557 (11)	−0.01868 (18)	0.68838 (4)	0.0683 (4)	

### Atomic displacement parameters (Å^2^)

**Table:**

	*U*^11^	*U*^22^	*U*^33^	*U*^12^	*U*^13^	*U*^23^
C1	0.066 (3)	0.076 (4)	0.072 (4)	0.002 (3)	0.013 (3)	−0.028 (3)
C2	0.049 (3)	0.043 (3)	0.049 (3)	−0.003 (2)	0.002 (2)	−0.007 (2)
C3	0.040 (2)	0.046 (3)	0.050 (3)	−0.002 (2)	0.003 (2)	0.000 (2)
C4	0.043 (3)	0.041 (3)	0.041 (3)	−0.003 (2)	−0.008 (2)	−0.002 (2)
C5	0.037 (2)	0.036 (2)	0.038 (2)	−0.004 (2)	−0.0005 (18)	0.008 (2)
C6	0.033 (2)	0.058 (3)	0.061 (3)	0.000 (2)	0.003 (2)	−0.007 (3)
C7	0.044 (3)	0.068 (4)	0.068 (3)	−0.007 (3)	−0.005 (2)	−0.021 (3)
C8	0.037 (2)	0.030 (2)	0.043 (2)	0.000 (2)	−0.0009 (19)	0.003 (2)
C9	0.044 (3)	0.032 (2)	0.043 (3)	0.001 (2)	0.001 (2)	0.004 (2)
C10	0.051 (3)	0.036 (2)	0.045 (3)	0.009 (2)	0.010 (2)	0.001 (2)
C11	0.043 (2)	0.029 (2)	0.044 (2)	0.004 (2)	0.005 (2)	−0.001 (2)
C12	0.046 (2)	0.046 (3)	0.049 (3)	−0.008 (2)	0.008 (2)	−0.001 (2)
C13	0.053 (3)	0.036 (3)	0.048 (3)	0.005 (2)	−0.012 (2)	0.000 (2)
C14	0.045 (3)	0.047 (3)	0.063 (3)	−0.009 (2)	0.005 (2)	0.020 (3)
C15	0.032 (2)	0.050 (3)	0.062 (3)	0.002 (2)	0.001 (2)	0.013 (2)
N1	0.039 (2)	0.043 (2)	0.049 (2)	0.0011 (19)	0.0011 (17)	−0.0014 (19)
N2	0.036 (2)	0.049 (2)	0.049 (2)	0.0006 (18)	0.0023 (16)	−0.0050 (19)
N3	0.042 (2)	0.054 (3)	0.054 (2)	0.000 (2)	0.0002 (19)	0.001 (2)
O1	0.061 (2)	0.098 (3)	0.072 (2)	−0.015 (2)	0.0116 (19)	−0.045 (2)
O2	0.0362 (16)	0.0386 (17)	0.0497 (19)	−0.0011 (14)	−0.0003 (13)	0.0021 (15)
S	0.0375 (6)	0.0510 (7)	0.0556 (7)	0.0047 (6)	0.0017 (5)	0.0007 (7)
Cl1	0.0728 (9)	0.0660 (9)	0.0661 (8)	−0.0008 (7)	−0.0169 (7)	0.0169 (7)

### Geometric parameters (Å, °)

**Table:**

C1—O1	1.404 (6)	C9—N2	1.282 (5)
C1—H1A	0.9600	C9—O2	1.361 (5)
C1—H1B	0.9600	C9—S	1.728 (4)
C1—H1C	0.9600	C10—C11	1.500 (5)
C2—O1	1.353 (5)	C10—S	1.816 (4)
C2—C3	1.381 (6)	C10—H10A	0.9700
C2—C7	1.375 (6)	C10—H10B	0.9700
C3—C4	1.378 (5)	C11—C15	1.379 (5)
C3—H3	0.9300	C11—C12	1.383 (6)
C4—C5	1.387 (5)	C12—N3	1.350 (5)
C4—H4	0.9300	C12—H12	0.9300
C5—C6	1.382 (5)	C13—N3	1.310 (5)
C5—C8	1.451 (5)	C13—C14	1.368 (6)
C6—C7	1.375 (6)	C13—Cl1	1.740 (4)
C6—H6	0.9300	C14—C15	1.373 (6)
C7—H7	0.9300	C14—H14	0.9300
C8—N1	1.289 (5)	C15—H15	0.9300
C8—O2	1.372 (4)	N1—N2	1.411 (4)
			
O1—C1—H1A	109.5	O2—C9—S	117.3 (3)
O1—C1—H1B	109.5	C11—C10—S	114.1 (3)
H1A—C1—H1B	109.5	C11—C10—H10A	108.7
O1—C1—H1C	109.5	S—C10—H10A	108.7
H1A—C1—H1C	109.5	C11—C10—H10B	108.7
H1B—C1—H1C	109.5	S—C10—H10B	108.7
O1—C2—C3	124.7 (4)	H10A—C10—H10B	107.6
O1—C2—C7	115.9 (4)	C15—C11—C12	117.2 (4)
C3—C2—C7	119.4 (4)	C15—C11—C10	121.5 (4)
C4—C3—C2	120.0 (4)	C12—C11—C10	121.3 (4)
C4—C3—H3	120.0	N3—C12—C11	123.9 (4)
C2—C3—H3	120.0	N3—C12—H12	118.1
C3—C4—C5	120.9 (4)	C11—C12—H12	118.1
C3—C4—H4	119.6	N3—C13—C14	124.7 (4)
C5—C4—H4	119.6	N3—C13—Cl1	116.2 (3)
C6—C5—C4	118.4 (4)	C14—C13—Cl1	119.0 (4)
C6—C5—C8	122.6 (4)	C13—C14—C15	118.2 (4)
C4—C5—C8	119.0 (4)	C13—C14—H14	120.9
C7—C6—C5	120.7 (4)	C15—C14—H14	120.9
C7—C6—H6	119.7	C14—C15—C11	119.6 (4)
C5—C6—H6	119.7	C14—C15—H15	120.2
C6—C7—C2	120.6 (4)	C11—C15—H15	120.2
C6—C7—H7	119.7	C8—N1—N2	106.8 (3)
C2—C7—H7	119.7	C9—N2—N1	105.7 (3)
N1—C8—O2	111.7 (4)	C13—N3—C12	116.3 (4)
N1—C8—C5	128.6 (4)	C2—O1—C1	118.4 (4)
O2—C8—C5	119.7 (4)	C9—O2—C8	102.5 (3)
N2—C9—O2	113.2 (4)	C9—S—C10	98.1 (2)
N2—C9—S	129.5 (4)		
			
O1—C2—C3—C4	179.6 (4)	C13—C14—C15—C11	−0.4 (7)
C7—C2—C3—C4	−3.0 (7)	C12—C11—C15—C14	2.6 (7)
C2—C3—C4—C5	0.6 (7)	C10—C11—C15—C14	−176.1 (4)
C3—C4—C5—C6	1.8 (6)	O2—C8—N1—N2	−0.1 (5)
C3—C4—C5—C8	−177.3 (4)	C5—C8—N1—N2	178.7 (4)
C4—C5—C6—C7	−1.8 (7)	O2—C9—N2—N1	0.2 (5)
C8—C5—C6—C7	177.2 (4)	S—C9—N2—N1	−179.1 (3)
C5—C6—C7—C2	−0.6 (7)	C8—N1—N2—C9	0.0 (4)
O1—C2—C7—C6	−179.4 (4)	C14—C13—N3—C12	1.6 (7)
C3—C2—C7—C6	3.0 (8)	Cl1—C13—N3—C12	−178.6 (3)
C6—C5—C8—N1	−171.9 (4)	C11—C12—N3—C13	0.9 (7)
C4—C5—C8—N1	7.2 (6)	C3—C2—O1—C1	−19.0 (7)
C6—C5—C8—O2	6.8 (6)	C7—C2—O1—C1	163.5 (5)
C4—C5—C8—O2	−174.1 (4)	N2—C9—O2—C8	−0.2 (4)
S—C10—C11—C15	−69.6 (5)	S—C9—O2—C8	179.1 (3)
S—C10—C11—C12	111.7 (4)	N1—C8—O2—C9	0.2 (4)
C15—C11—C12—N3	−3.0 (7)	C5—C8—O2—C9	−178.7 (3)
C10—C11—C12—N3	175.8 (4)	N2—C9—S—C10	−2.2 (5)
N3—C13—C14—C15	−1.8 (8)	O2—C9—S—C10	178.6 (3)
Cl1—C13—C14—C15	178.4 (4)	C11—C10—S—C9	−79.9 (3)
